# Supplementary material for: Ras-Related Protein Rab-32 and Thrombospondin 1 Confer Resistance to the EGFR Tyrosine Kinase Inhibitor Osimertinib by Activating Focal Adhesion Kinase in Non-Small Cell Lung Cancer
Source: Cancers (Basel). 2022 Jul 14;14(14):3430. doi: 10.3390/cancers14143430 (PMC9317954; doi:10.3390/cancers14143430)
Supplement: Supplementary file 1 [file cancers-14-03430-s001.zip › cancers-1760185-supplementay.pdf]

# Supplementary Materials: Ras-Related Protein Rab-32 and Thrombospondin 1 Confer Resistance to the EGFR Tyrosine Kinase Inhibitor Osimertinib by Activating Focal Adhesion Kinase in Non-Small Cell Lung Cancer

**Zeinab Kosibaty, Odd Terje Brustugun, Inger Johanne Zwicky Eide, Georgios Tsakonas, Oscar Grundberg, Luigi De Petris, Marc McGowan, Per Hydbring and Simon Ekman**

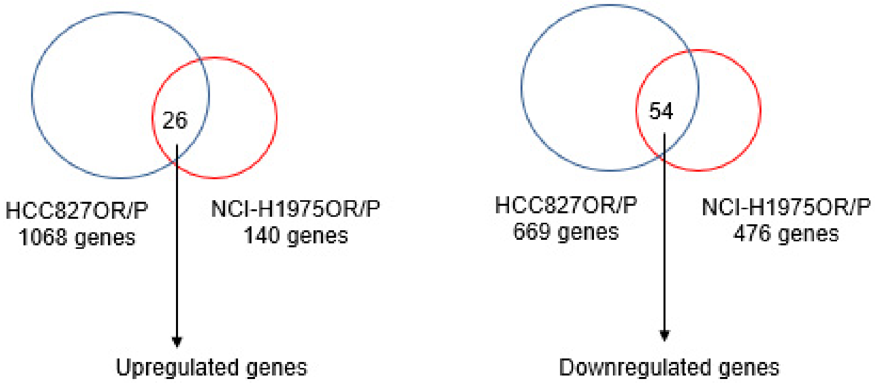


**Figure S1.** siRNA-library screening in HCC827OR and HCC827P cells. Knockdown of 24 genes in HCC827OR (upper panel) and HCC827P (lower panel) using 3 sets of siRNAs for 72 h. Each bar visualizes cell viability for a given siRNA in comparison to cells transfected with a scrambled negative control. siRNAs specific for the GAPDH gene were employed as a positive control. Error bars represent the mean ± SD from three independent experiments.

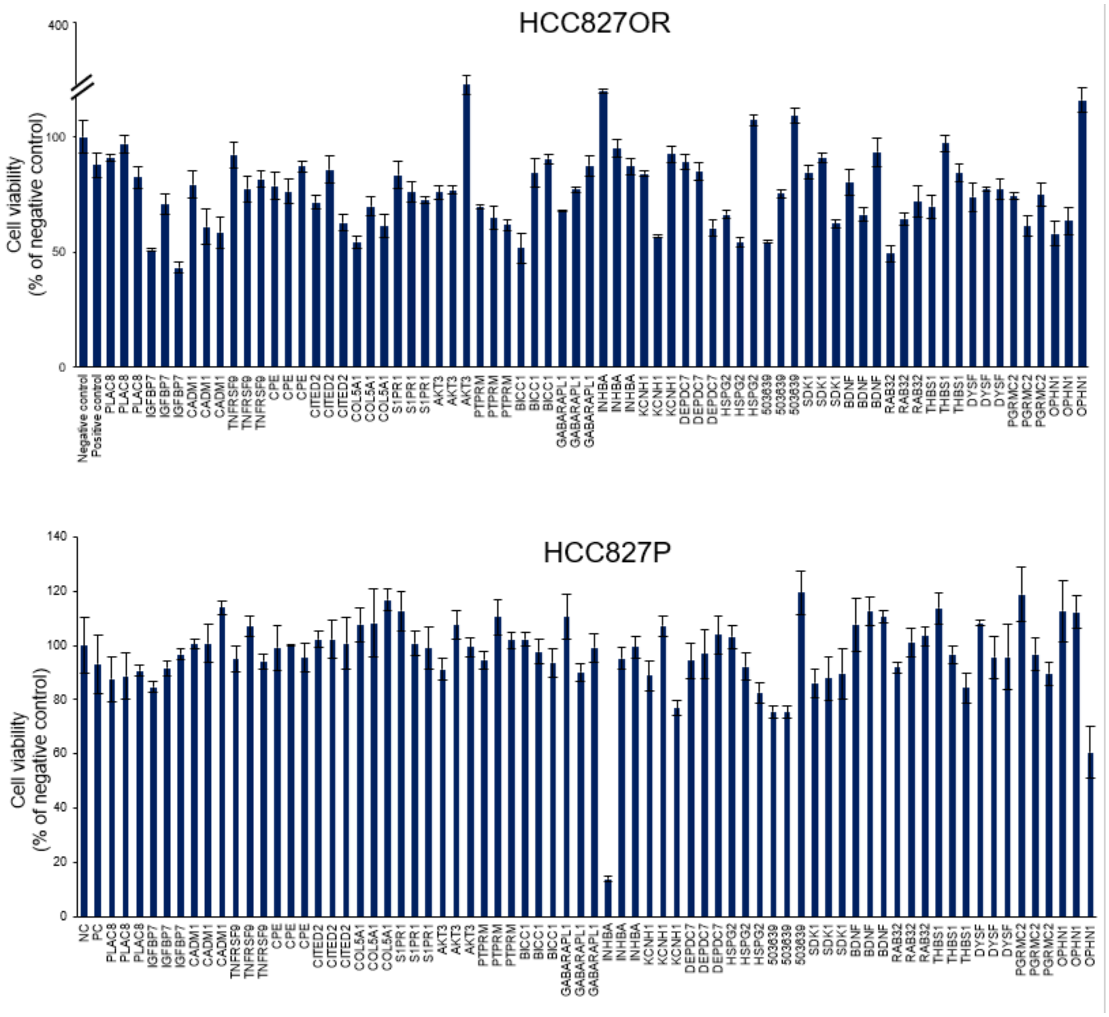


**Figure S2.** siRNA-library screening in NCI-H1975OR and NCI-H1975P cells. Knockdown of 24 genes in NCI-H1975OR (upper panel) and NCI-H1975P (lower panel) using 3 sets of siRNAs for 72 h. Each bar visualizes cell viability for a given siRNA in comparison to cells transfected with a scrambled negative control. siRNAs specific for the GAPDH gene were employed as a positive control. Error bars represent the mean ± SD from three independent experiments.

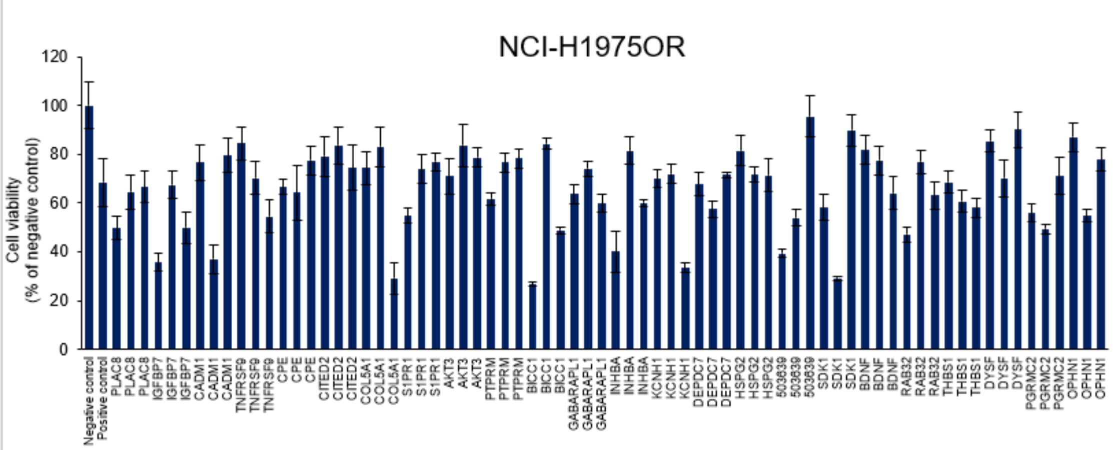


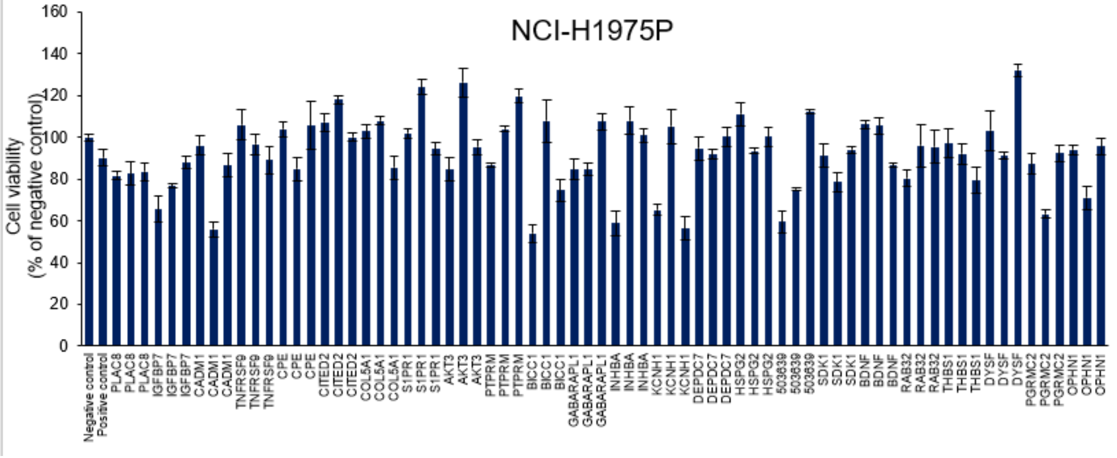


**Figure S3.** Venn diagram showing 26 upregulated and 54 downregulated overlapping genes in HCC827OR/P and NCI-H1975OR/P.

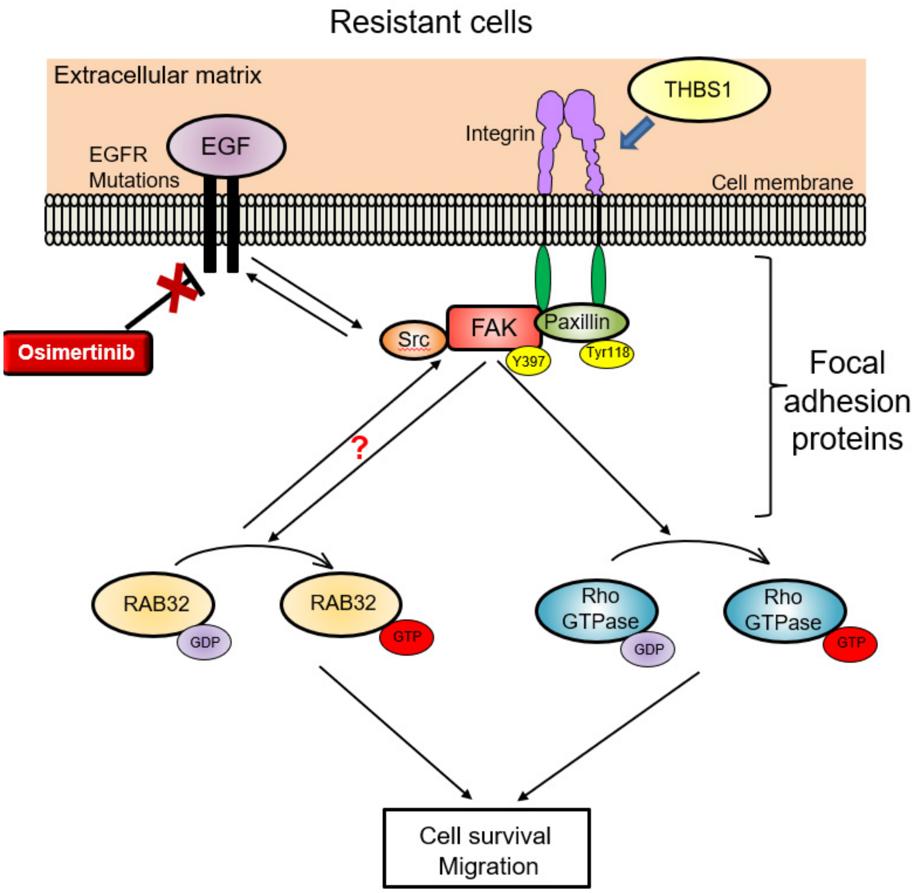


**Figure S4.** Model for RAB32-THBS1-mediated EGFR and FAK signaling in osimertinib resistance of NSCLC cells. Overexpression of THBS1 in osimertinib resistance promotes FAK autophosphorylation at Y397 and phosphorylation of paxillin at Tyr118. The activation of FAK signaling enhances Rho small GTPase and downstream oncogenic functions. Additionally, RAB32, a small GTPase, is potentially enhanced via activation of a FAK signaling complex. Overexpression of RAB32 in osimertinib resistance likely provides a feedback signal to activate FAK signaling. It is established that EGFR can activate Src and, vice versa, that Src activates EGFR. EGFR and FAK signaling could simultaneously enhance osimertinib resistance leading to promotion of cell growth and migration.

Figure 1C

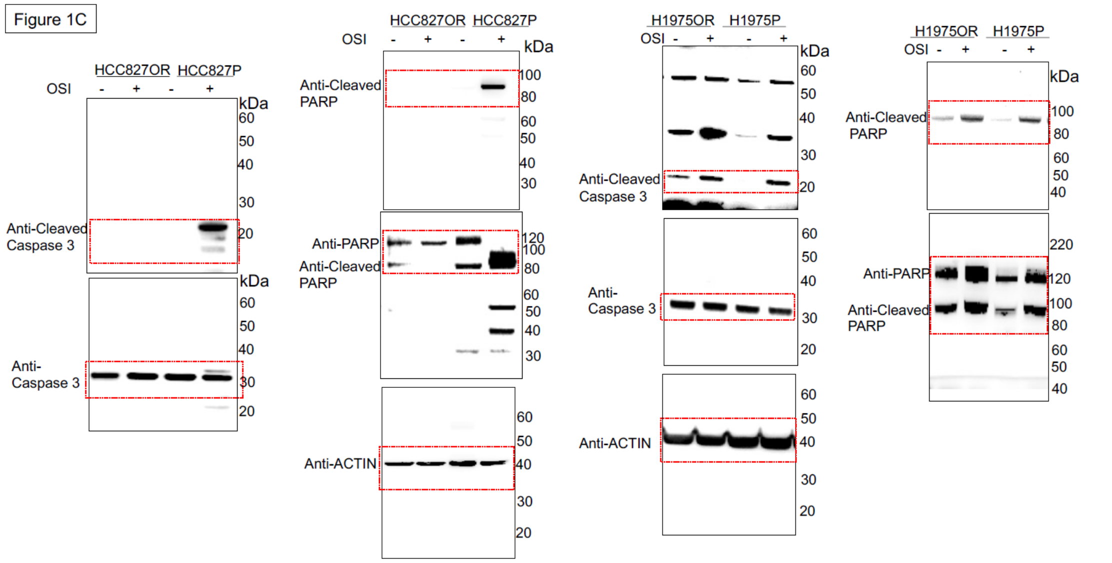


Figure 2B

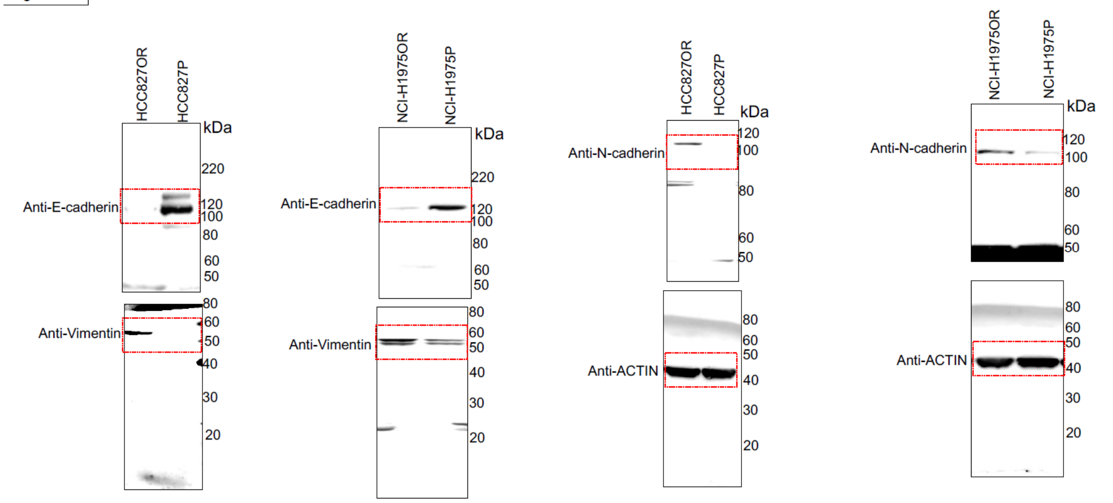

Figure 5B

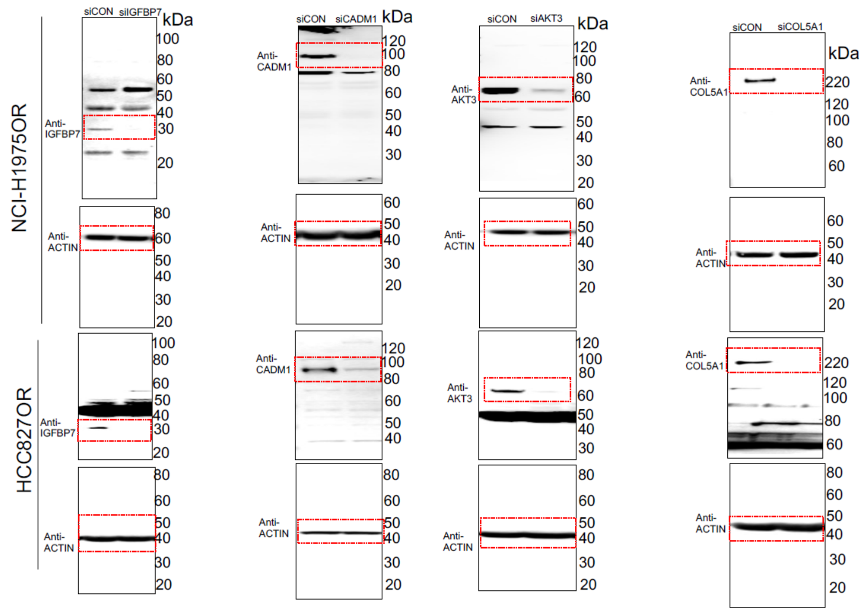


Figure 5B continue

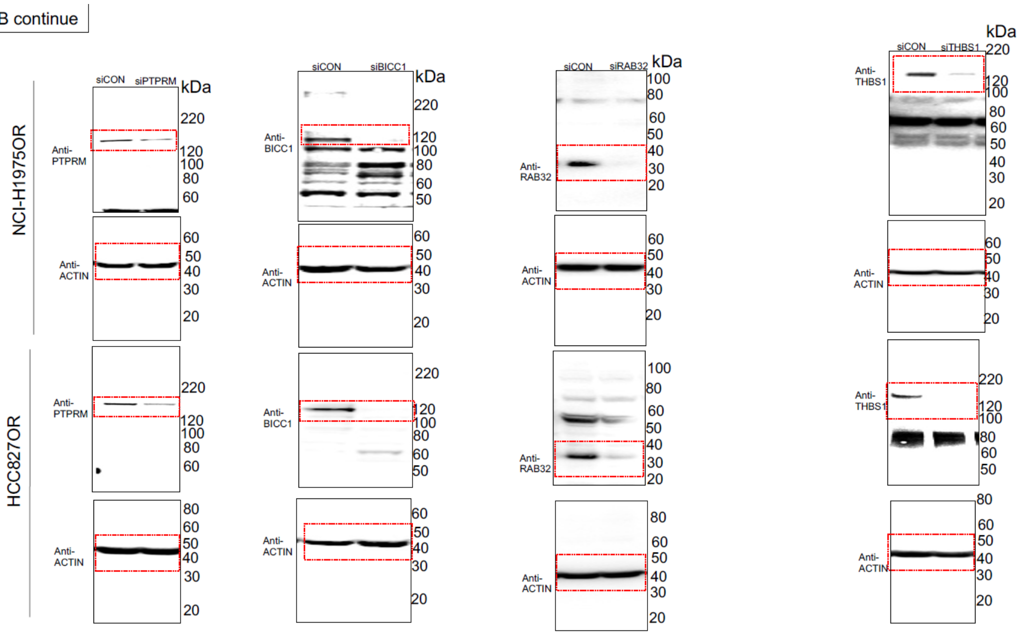

Figure 7B

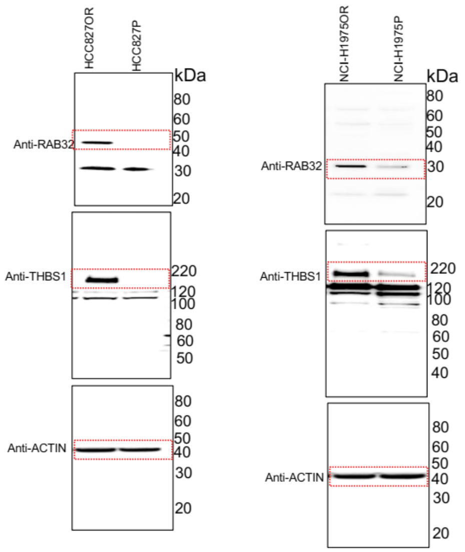


Figure 7C

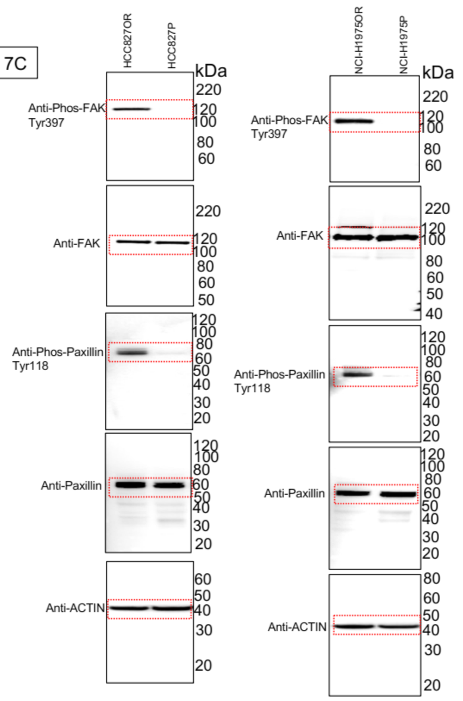


Figure 7D

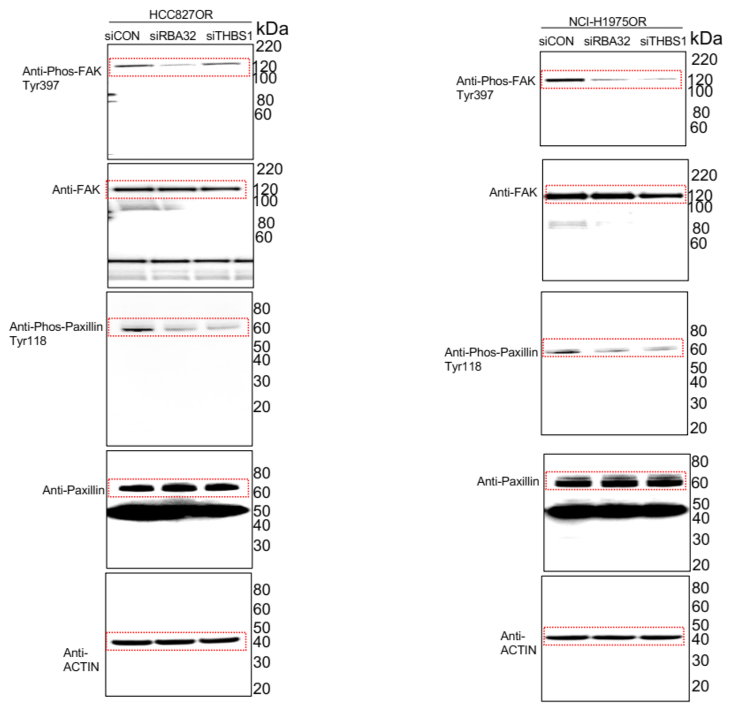

Figure 7E

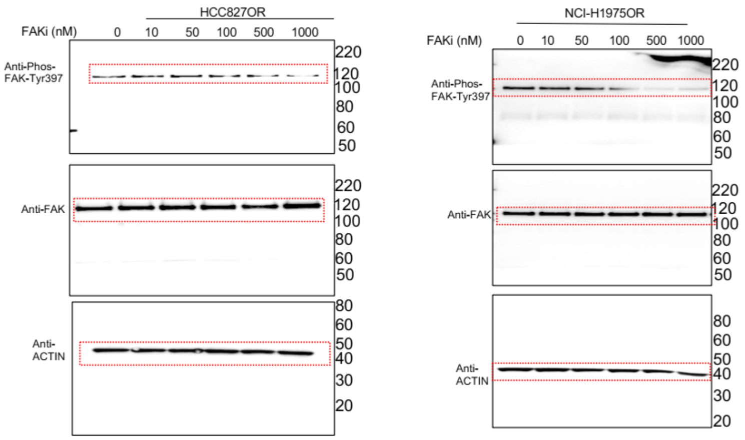


**Figure S5.** full-length of Western blots.
